# Spigelian-Cryptorchidism Syndrome: A Literature Review and Case Report of a Rare Clinical Entity

**DOI:** 10.7759/cureus.87324

**Published:** 2025-07-05

**Authors:** Hamdah T Kalantar, Hussein Naji, Abdulla Naji

**Affiliations:** 1 Medicine and Surgery, Mohammed Bin Rashid University of Medicine and Health Sciences (MBRU), Dubai, ARE; 2 Pediatric Surgery, Mediclinic Parkview Hospital, Dubai, ARE; 3 Internal Medicine, Skaraborgs Sjukhus Skövde, Skövde, SWE

**Keywords:** congenital hernia, cryptorchidism, intra-abdominal testis, neonatal abdominal wall defect, pediatric hernia, rare pediatric syndrome, spigelian-cryptorchidism syndrome, spigelian fascia, spigelian hernia, undescended testis

## Abstract

Spigelian-cryptorchidism syndrome is a rare and diagnostically challenging condition in neonatology that is characterized by the association of an undescended testis with a Spigelian hernia, the latter usually without radiologic evidence. We report the case of a full-term male neonate delivered at 39 weeks and two days of gestation by kiwi-assisted vaginal delivery after a spell of fetal bradycardia. Antenatal imaging had shown persistent bilateral pelviectasis of the kidneys and an undescended right testis. Postnatally, the initial course was normal; however, clinical evaluation showed partially reducible sausage-shaped swelling in the right lower quadrant and an impalpable right testis. Ultrasonographic examination confirmed intra-abdominal location of the right testis and showed bilateral mild dilatation of the renal pelvis but no abdominal wall defect or hernia sac. Surgical assessment revealed a partially reducible mass, consistent with Spigelian hernia, and hence established the setting of Spigelian-cryptorchidism syndrome. The testis's anatomical separation from the inguinal canal and the lack of a discernible processus vaginalis favored a syndromic diagnosis. The case brings to light the inherent limitations of imaging in the detection of neonatal Spigelian hernia, the value of physical examination by skilled clinicians, and the imperative of a multidisciplinary diagnostic and therapeutic approach. Conservative monitoring was chosen, with surgical repair scheduled at 12 months of age, consistent with best practices at present. This case supports that in neonatal presentations with radiological uncertainty combined with strong clinical evidence, the diagnosis can be pursued critically and not delayed based on imaging. It also stresses the requirement to consider unexplained abdominal distension and non-palpable testes on a differential which includes this uncommon but clinically important syndrome.

## Introduction

Spigelian hernia is an uncommon congenital or acquired defect along the semilunar line, where the transversus abdominis aponeurosis becomes the rectus sheath. It forms less than 2% of abdominal wall hernias and is rarely found in neonates, so early detection is especially difficult [1,2]. The congenital type usually has an insidious presentation and is usually not discovered until complications have developed. Because of its rarity, occurrence in the pediatric population is unreported, but less than 100 pediatric cases have been described globally [3].

A particular condition under this category, Spigelian-cryptorchidism syndrome, is the occurrence of a Spigelian hernia along with ipsilateral undescended testis, most commonly on the right side [4]. The combination, although uncommon, has been reported sufficiently to indicate a clinically significant pattern instead of coincidence [5]. Raveenthiran has suggested that the hernia serves as either a mechanical block or a diversion tract, and the testis becomes "stuck" within the hernia sac or does not descend at all [4]. The Spigelian fascia defect is wide enough to allow the testis to herniate into the sac, causing diagnostic uncertainty with other inguinal hernias. Imaging and intraoperative presentations in several case reports reveal intra-abdominal or ectopic testicular positions near the Spigelian defect [4,6,7]. The hernia may not produce a visible bulge in neonates, and ultrasonographic findings are often inconclusive, especially in the presence of overlying bowel gas [7,8]. Moreover, in the absence of an obvious abdominal wall defect, the presence of a non-palpable testis may divert attention solely toward urological causes, delaying surgical consultation [9,10]. This report presents a rare case of Spigelian-cryptorchidism syndrome in a term male neonate with a right intra-abdominal testis and a partially reducible mass in the right iliac fossa. We aim to critically highlight the importance of clinical suspicion in the absence of definitive imaging, discuss this syndrome's embryological and anatomical underpinnings, and outline a rational approach to diagnosis and management in early infancy.

## Case presentation

A full-term male neonate was born at 39 weeks and two days of gestation to a G2P2 mother via a kiwi-assisted vaginal delivery, prompted by a concerning episode of fetal bradycardia. The mother, with a blood group of O positive, had an unremarkable serologic profile for antenatal infections, and high vaginal swab screening for group B *Streptococcus* was negative. Routine obstetric surveillance had noted persistent bilateral renal pelviectasis and a right intra-abdominal undescended testis on antenatal imaging.

The neonate had reassuring APGAR scores of 8 and 9 at one and five minutes, respectively, and a birth weight of 3.34 kg. He measured 50 cm in length, and his head circumference was 34.5 cm. The baby cried at birth and required no active resuscitation beyond routine newborn care. Skin-to-skin contact with the mother was initiated shortly after delivery.

**Figure 1:**
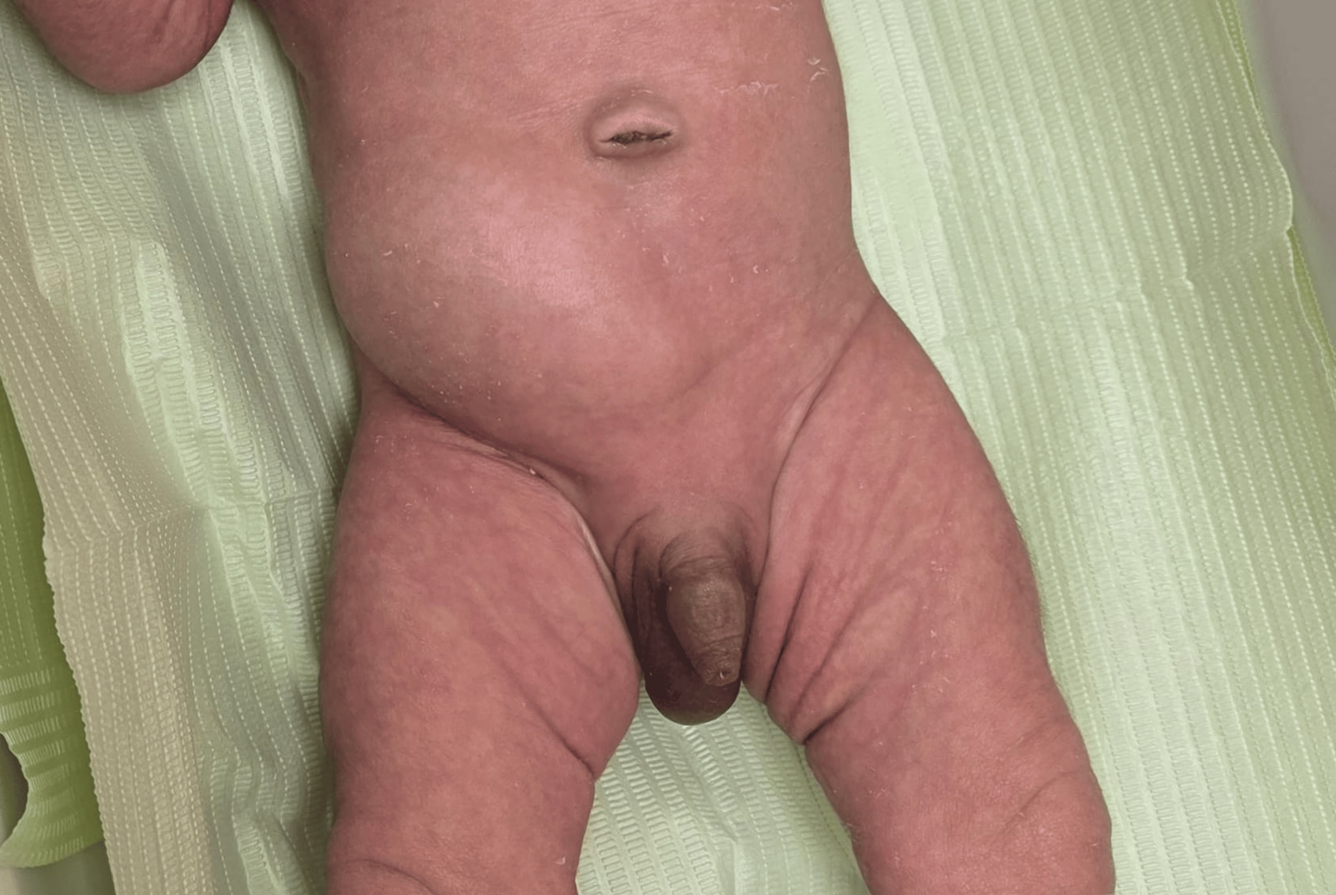
Spigelian hernia with an undescended right testis in our patient

Despite a favorable immediate postnatal course, physical examination revealed a number of significant clinical findings that warranted closer evaluation. On general examination, he appeared well and alert. Notably, there was a mild form of tongue tie. The cardiovascular system was clinically normal with normal heart sounds and no murmurs detected at birth. Femoral pulses were bilaterally palpable. Respiratory assessment was unremarkable with equal bilateral air entry. The abdomen was soft, non-distended, and without palpable organomegaly, but there was a cylindrical, sausage-shaped swelling in the right lower quadrant, measuring approximately 8×5 cm, which became more pronounced with crying. The swelling was partially reducible, suggesting a dynamic lesion (Figure 1). Genital examination confirmed a right non-palpable undescended testis and a retractile left testis. The back, spine, and hips were normal with no signs of click or deformity. A summary of the clinical findings is provided in Table 1 for clarity.

**Table 1:** Summary of clinical findings USG: ultrasound

Parameter	Observation
Gestational age	39+2 weeks
Mode of delivery	Kiwi-assisted vaginal delivery due to fetal bradycardia
APGAR scores	8 at 1 minute and 9 at 5 minutes
Birth weight	3.34 kg
Length	50 cm
Head circumference	34.5 cm
General appearance	Well, alert, mild posterior tongue tie
Eyes	Red reflex present bilaterally
Cardiovascular	S1, S2 present, no murmur, bilateral femoral pulses palpable
Respiratory	Equal air entry bilaterally
Abdomen	Soft, non-distended, normal on palpation
Genitalia	Right undescended testis (intra-abdominal), left retractile testis
Spine/back	Normal
Hips	No click detected
Abdominal swelling	8×5 cm cylindrical mass in the right lower quadrant, partially reducible, increases with crying
Postnatal USG of the testis	Right testis intra-abdominal, in the right iliac fossa; left testis retractile
USG of the abdomen	Mild bilateral renal pelviectasis (right: 5.4 mm; left: 4.4 mm); otherwise, normal kidneys and bladder
Clinical examination	Sausage-shaped mass (4×6 cm) in the right iliac fossa; soft, reducible testis not palpable on the right side

Imaging and diagnosis 

Given the antenatal findings and clinical signs, postnatal ultrasonographic evaluation was promptly undertaken at day 1 of age. Ultrasound of the testes confirmed that the right testis was undescended and located intra-abdominally in the right iliac fossa. The left testis was retractile but otherwise unremarkable.

**Figure 2:**
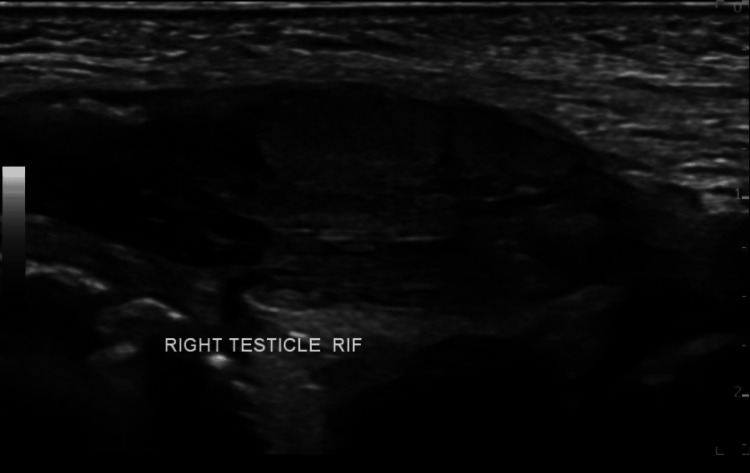
Ultrasound image showing the right testis in the right iliac fossa The scan demonstrates an ovoid, homogeneously hypoechoic structure consistent with testicular tissue, located intra-abdominally. This is indicative of an undescended right testis. RIF: right iliac fossa

Ultrasonographic examination of the kidneys, ureters, and bladder revealed that the right kidney measured 4.9×2 cm with a cortical thickness of 0.5 cm and the anteroposterior diameter of the renal pelvis was 8.5 mm. The left kidney measured 4.7×2.5 cm, also with a cortical thickness of 0.5 cm and a renal pelvic diameter of 6.8 mm. Both kidneys appeared normal in size, echotexture, and cortical-medullary differentiation. The urinary bladder was adequately distended with no evidence of wall thickening, intravesical calculi, or debris. The right testis was visualized in the right iliac fossa, consistent with an intra-abdominal undescended testis (Figure 2). No abdominal mass was detected, and no facial defect was identified.

A pediatric surgical evaluation on day 2 of age confirmed the presence of an 8×5 cm soft, partially reducible, sausage-shaped mass in the right iliac region, accentuated during crying. The right testis remained impalpable, while the left was easily felt in the scrotum.

**Figure 3:**
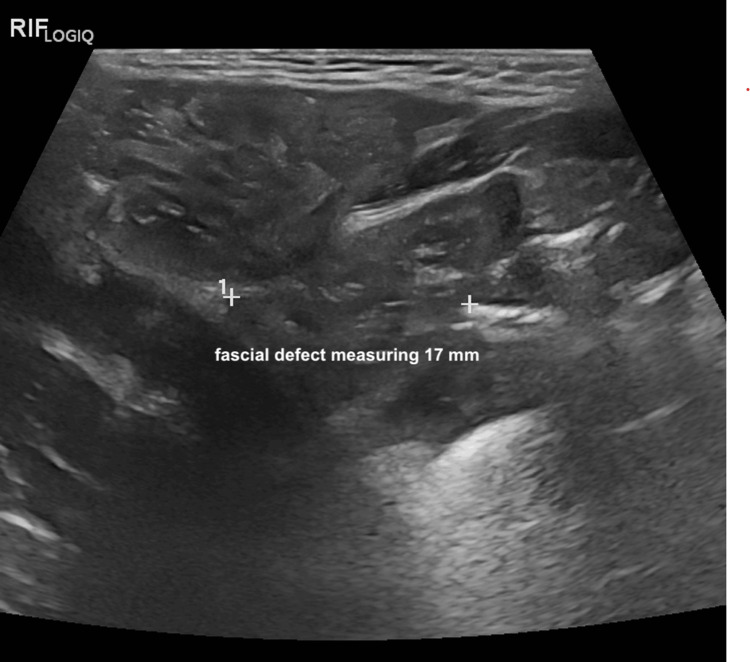
Ultrasound of the abdominal wall showing a fascial defect consistent with hernia Ultrasound of the right iliac fossa demonstrates a 17 mm fascial defect suggestive of an abdominal wall hernia, with possible herniation of fat or bowel through the defect. RIF: right iliac fossa

On day 3 of life, a repeat ultrasound of the abdominal wall was conducted with a focused assessment for Spigelian hernia. The scan revealed a 17 mm defect in the right lateral aspect of the anterior abdominal wall, through which intraperitoneal contents were observed protruding (Figure 3). These findings were in keeping with a Spigelian hernia, with the herniated sac measuring approximately 60×22 mm. No free intraperitoneal fluid was detected. These sonographic findings were consistent with bilateral hydronephrosis, an intra-abdominal undescended testis, and a large ventral Spigelian hernia on the right side.

Management plan

Taking into account the unusual location of the undescended testis in conjunction with the abdominal wall swelling, the clinical picture aligned with a rare but well-recognized entity: Spigelian-cryptorchidism syndrome. This condition, also referred to in literature as the Spigelian hernia-associated undescended testis syndrome, is characterized by an ectopic testis associated with a Spigelian hernia, although a definitive hernia sac may not always be demonstrable on imaging in neonates due to gas artifact or reducibility of the mass.

The management plan, after thorough multidisciplinary discussion, was to observe conservatively. The consensus was to operate when the child is one year of age. Elective surgeries in children, particularly infants, are often postponed until around one year of age due to a combination of medical, physiological, and developmental factors [11]. Neonates and young infants are more sensitive to anesthetics, increasing the risk of cardiovascular and neurological side effects [12].

## Discussion

The diagnostic pathway in cases of suspected Spigelian-cryptorchidism syndrome in the neonatal period demands scrutiny that extends well beyond routine imaging and clinical observation. In current neonatal practice, ultrasound remains the initial modality for evaluating undescended testes and abdominal wall anomalies due to its safety, accessibility, and absence of ionizing radiation [13]. However, its diagnostic yield in the context of Spigelian hernias is notably limited, particularly when the fascial defect is minute, the hernia contents are non-bowel in nature, or the defect intermittently presents with no visible sac [14]. In the present case, the first ultrasonography study successfully localized the right testis to an intra-abdominal position and documented mild bilateral renal pelviectasis, but it failed to identify a fascial defect or convincing evidence of herniation. This is not a limitation of technique or operator, but rather a reflection of the anatomical and physiological subtleties inherent in neonatal Spigelian hernias [15]. These hernias often emerge through a narrow, transverse defect in the Spigelian fascia, and unlike classical inguinal hernias, they may not produce a protruding sac or evident peritoneal communication [16]. Moreover, the transient nature of the herniation, often exacerbated by crying or straining, may escape detection during a static imaging session. Consequently, the over-reliance on ultrasonography in this context can easily result in underdiagnosis or misdiagnosis, especially if the radiological findings are interpreted without reference to nuanced clinical signs [16].

Advanced imaging, such as MRI or CT, may offer higher spatial resolution and superior delineation of the muscular and fascial planes, and in older patients or in complex cases, they may clarify equivocal findings. However, their role in the neonatal setting remains limited due to logistical challenges such as the need for sedation and positioning and because their routine use is neither practical nor justifiable in most clinical settings. Furthermore, these modalities are often reserved for atypical or persistent cases where initial interventions have failed or the anatomy remains unclear. Thus, the cornerstone of diagnosis in Spigelian-cryptorchidism syndrome remains the clinical examination, particularly the surgeon's hands and experienced judgment [17]. In this case, the key diagnostic clue, that is, a soft, sausage-shaped swelling in the right lower quadrant that became more prominent with crying, could not have been captured adequately on imaging. The partial reducibility of the mass and its persistence in the same anatomical location, even in the absence of an overt fascial defect on ultrasound, were highly suggestive of an occult Spigelian hernia. This highlights a crucial clinical principle: the absence of radiological evidence does not negate the presence of a pathological entity, particularly when physical examination findings are unequivocal. In neonatal surgery, where anatomical presentations are often embryologically deviant and not constrained by textbook expectations, the clinician's interpretive skill is not supplementary to imaging; it is superior to it.

The coexistence of a Spigelian hernia with cryptorchidism raises deeper embryological questions, none of which have yet received a universally accepted answer. Several pathophysiological theories attempt to explain this association, each with its merits and deficiencies. One hypothesis proposes a developmental arrest during the critical phases of abdominal wall closure and testicular descent. According to this view, a disruption in the mesodermal layer results in both a weakened Spigelian zone, predisposing to herniation, and a failure in the downward migration of the testis [18]. An alternative theory suggests that the testis, while descending, may be diverted along an aberrant path by a preexisting fascial defect, thereby bypassing the inguinal canal entirely. This could explain the frequent absence of a processus vaginalis in these cases and the localization of the testis in the iliac fossa rather than the scrotum or inguinal canal. Another possibility is that the herniation occurs first and physically impedes the descent of the testis, essentially trapping it intra-abdominally and preventing normal gubernacular traction. A more provocative interpretation is that this syndrome may in fact represent a variant of testicular ectopia, wherein the testis migrates outside its normal anatomical course, not as a consequence of mechanical obstruction but due to a misdirection of the guiding structures themselves. While the true embryological sequence remains unsettled, what is evident is that the testicular malposition is not merely incidental but likely integral to the hernia's pathogenesis or vice versa. The observed anatomical anomaly is not dual pathology; it is a singular developmental misrouting with two manifestations [19].

Management of Spigelian-cryptorchidism syndrome in the neonatal period must be deliberate and, above all, contextual. Immediate surgical intervention is not routinely indicated in the absence of acute complications such as incarceration or strangulation. The prevailing approach advocates for observation during the early months of life, with elective surgical correction planned between six months and one year of age. This timing is informed by multiple considerations: optimizing testicular viability, minimizing anesthesia risks, and allowing for growth that facilitates surgical handling. The notion that early surgery ensures better long-term fertility outcomes is supported by testicular biology, but must be weighed against the potential hazards of neonatal anesthesia and the increased technical difficulty posed by smaller anatomical landmarks.

When surgery is undertaken, it must address both the hernia and the undescended testis. The choice between open and laparoscopic approaches should be individualized. Laparoscopy offers the advantage of direct visualization of intra-abdominal anatomy and can be diagnostic as well as therapeutic. It is especially useful when the testis is not palpable or when the hernia is not well localized. However, in cases where the mass is externally appreciable and the fascial defect is suspected to be superficial, open repair may be equally effective. The surgical objective is twofold: to reduce the hernia and to mobilize and fix the testis within the scrotum without tension. Identification and closure of the hernia sac, if present, is essential, though in many Spigelian cases, the sac may be rudimentary or absent altogether. Intraoperative findings often defy radiological expectations, further emphasizing the inadequacy of imaging alone in guiding management.

A critical consideration is the potential risk of hernia incarceration if surgical correction is deferred. Although Spigelian hernias are less prone to incarceration than their inguinal counterparts, the presence of a persistent, reducible mass in an infant still carries a measurable risk. Moreover, the uncertainty in the structural integrity of the fascial planes, combined with the testis being an atypical hernia content, compounds the potential for ischemic complications. Postoperative follow-up is imperative, not only to confirm testicular viability and scrotal positioning but also to monitor for recurrence and evaluate renal function. In this case, the antenatal and postnatal renal findings, although currently non-obstructive, require ongoing surveillance given the association between cryptorchidism and urinary tract anomalies.

The mild bilateral renal pelviectasis observed antenatally and on the initial day 1 ultrasound likely represents an early stage of urinary tract dilation that became more definitively evident as moderate bilateral hydronephrosis on the day 3 scan. This change may indicate either true progression or improved detection due to optimized imaging conditions. Possible causes include transient urinary tract obstruction, external compression from the ventral hernia, or associated congenital anomalies linked with cryptorchidism. Currently, no definitive ureteral obstruction has been identified on imaging. Given the risk of progression, close follow-up with serial renal ultrasounds is essential to monitor for any worsening dilatation or development of obstructive uropathy. If hydronephrosis persists or worsens, further functional imaging, such as a MAG3 renal scan, may be warranted to assess renal drainage and guide management decisions.

Furthermore, the concurrent presence of cryptorchidism, renal tract anomalies, and abdominal wall defects suggests a shared embryological origin involving the disruption of mesodermal and intermediate mesodermal development. This developmental misrouting likely contributes to the complex phenotype observed rather than representing isolated anomalies.

Regarding surgical management, laparoscopic repair offers advantages including enhanced visualization of intra-abdominal structures, minimally invasive access, and reduced postoperative pain. Importantly, evidence suggests laparoscopy may reduce the risk of postoperative adhesions compared to open laparotomy, potentially decreasing long-term complications such as bowel obstruction or infertility. Nevertheless, the choice between laparoscopic and open approaches should be individualized based on patient-specific factors, surgeon expertise, and resource availability.

The literature surrounding Spigelian-cryptorchidism syndrome remains limited, with reported cases numbering fewer than 60 and true neonatal diagnoses constituting only a small fraction. Most cases are discovered incidentally during exploration for non-palpable testes or present later with complications from undiagnosed hernias. Several patterns emerge from the existing reports. The condition shows a marked right-sided predominance and often presents without a demonstrable hernia sac. In many cases, imaging is non-diagnostic, and the diagnosis is made intraoperatively. These observations emphasize the need to keep a high index of suspicion, especially in neonates who present with unexplained abdominal masses and non-palpable testes [20]. Notably, the diagnosis and management of the syndrome almost always need to be achieved with a multidisciplinary effort, including neonatology, pediatric surgery, radiology, and occasionally pediatric urology. This cooperative paradigm guarantees that subtle observations are not ruled out and that infrequent syndromic links are not lost in the haste to reclassify pathology into more common language. The clinical acuity, anatomical interest, and surgical readiness needed in such instances single them out as educational not merely due to their uniqueness but because of the precision and delicacy they necessitate at each stage of treatment. A summary of key diagnostic and clinical features across reported cases is presented in Table 2 to illustrate the variability in presentation and diagnostic yield.

**Table 2:** Comparative overview of reported cases of Spigelian-cryptorchidism syndrome US: ultrasound; RLQ: right lower quadrant

Author (year)	Study type/no. of cases	Age/side	Key clinical features	Imaging findings	Surgical approach	Main takeaway
Berlin and Meyers, 2019 [13]	Neonatal imaging review	Neonatal/N/A	Imaging guidelines for undescended testes and wall defects	US preferred; limited in small fascial defects	N/A	Clinical context must guide imaging interpretation
Jones and Hutson, 2015 [14]	Literature review/40 cases	0-4 years/mostly right	Non-palpable testis±mass	US often non-diagnostic	Mostly open	Syndromic link proposed; high index of suspicion needed
Adil et al., 2023 [15]	Case report/1 case	Neonate/right	Soft, reducible RLQ mass+intra-abdominal testis	US missed hernia; localized testis	Open repair+orchidopexy	Imaging unreliable; clinical signs critical
Kwee and Kwee, 2018 [16]	Meta-analysis	N/A/N/A	Clinically occult groin hernias	US sensitivity low in subtle hernias	N/A	Static US alone often insufficient
Toms et al., 2002 [17]	Pictorial review	Pediatric-adult/variable	Various abdominal hernias	MRI/CT superior to US	N/A	Advocates cross-sectional imaging when US is inconclusive
Montero and Rico-Jimenez, 2025 [18]	Review/52 cases	Birth-adolescence/~80% right	Abdominal mass+cryptorchidism	US often equivocal; MRI/CT more definitive	Laparoscopy used in 28%	Favors embryological misrouting theory
Durham and Ricketts, 2006 [19]	Case series/3 cases	Neonates/right	Non-palpable testis+mass	US failed in all cases	Open repair	Supports developmental arrest theory
Farina et al., 2024 [20]	Case report/1 case	Infant/right	Palpable RLQ mass+testicular maldescent	CT diagnostic; US non-contributory	Laparoscopy	Stresses need for combined clinical+imaging strategy

## Conclusions

Spigelian-cryptorchidism syndrome is still a diagnostically tricky but clinically significant entity, particularly in the neonatal period when its subtle physical features can readily be missed and imaging often falls short. Although today's management is in favor of elective repair at the age of six months to one year, individualized decision-making with a multidisciplinary team remains essential. Future research directions would focus on optimizing imaging protocols for neonatal abdominal wall assessment, such as dynamic ultrasound methods and improved cross-sectional imaging in carefully selected cases. Concurrently, the creation of a centralized database for Spigelian-cryptorchidism syndrome could allow for data aggregation, enhance the characterization of anatomical variations, and enable evidence-based guidelines for the diagnosis and timing of intervention. Additional embryologic and molecular research may also elucidate pathogenesis, leading to earlier detection and possibly preventive intervention.
